# Does the delivery of diagnostic news affect the likelihood of whether or not patients ask questions about the results? A conversation analytical study

**DOI:** 10.1111/hex.12693

**Published:** 2018-05-03

**Authors:** Ged M. Murtagh, Anne L. Thomas, Lynn Furber

**Affiliations:** ^1^ Division of Surgery Department of Surgery and Cancer St Mary's Campus Imperial College London London UK; ^2^ Department of Cancer Studies and Molecular Medicine Leicester Royal Infirmary Leicester UK; ^3^ School of Nursing and Midwifery Edith Murphy De Montfort University Leicester UK

**Keywords:** conversation analysis, diagnostic news announcements, oncology, patient question asking

## Abstract

**Background:**

Asymmetries in knowledge and competence in the medical encounter often mean that doctor‐patient communication can be compromised. This study explores this issue and examines whether the likelihood of patient question asking is increased following the delivery of diagnostic test results. It also examines whether that likelihood is related to the way in which the test results are delivered.

**Objective:**

To examine when and how patients initiate questions following diagnostic news announcements.

**Methods:**

We audio‐recorded oncology consultations (n = 47) consisting of both first consultations and follow‐up consultations with patients with different types of cancer, at a leading UK teaching hospital. From the primary sample, we identified 30 consultations based on a basic count of the frequency of patient questions and their positioning in relation to diagnostic announcements. This subset of 30 consultations consisted of a mix of first and follow‐up consultations.

**Results:**

Our data demonstrate how the design and delivery of diagnostic news announcements can either discourage or provide the opportunity for a patient‐initiated question in the next turn of talk. We identified two types of announcement. Q+ generally provided for a patient‐initiated question as a relevant next turn following the news announcement, whereas Q− did not. Q+ was sometimes followed up with the explanation of test results, which appeared to encourage further patient questions.

**Conclusion:**

The design and delivery of diagnostic news announcements can make a patient‐initiated question more or less appropriate, in the next turn of talk. In addition, showing and explaining test results can encourage further opportunities for patients’ questions.

## 1 INTRODUCTION

Clinician‐patient communication is an integral feature of the doctor‐patient relationship. The character of that communication can, either directly or indirectly, influence health outcomes.^1^, ^2^ For example, when the communication results in higher levels of patient involvement, patients assume a greater sense of control over their health and consequently are more likely to comply with treatment recommendations.^2^, ^3^, ^4^ However, patient involvement in the consultation can include different types of communication behaviours, all of which can be shaped by the setting and the activities within that setting.^3^, ^5^

Asking questions is one of the primary ways in which patients involve themselves in the consultation process.^3^ This simple verbal act is also linked directly to patients’ information needs^6^ as well as improved information provision from clinicians^3^, ^7^ and more recently has been demonstrated as a powerful intervention to shape physician behaviour.^8^ However, the opportunities for a patient to ask a question in the consultation may not always be maximized. Even when patients have strong preferences for information (to be provided), this does not always result in patients engaging in information‐seeking behaviours, like question asking.^6^ In part, this may be due to the specific structure of the medical encounter^9^ where typically doctors lead the sequence of activities (information gathering, history taking, differential diagnosis, treatment proposals, etc.) to be accomplished within the consultation. This routinized structure is so embedded in conventional understandings of the doctor‐patient relationship that typically patients intuitively comply with it,^10^ often surrendering the expression of communication behaviours like question asking. Frankel^9^ provides some telling evidence on this issue. Using findings from early conversation analytical studies of doctor‐patient encounters, he argued that the turn‐taking system found in the medical interview exhibits a much more restrictive interactive form when compared to ordinary interaction. This form is shaped by the doctor's objectives and interests, which typically imposes constraints that deter, rather than encourage, patient involvement. The result is that “routine restrictions [are] placed upon speakers and the types of turn organizational formats they conventionally employ.” Consequently, Frankel argues, patient‐initiated direct questions rarely appear in the medical encounter. When they do appear, he suggests, they do so with some form of subtle modification.

Dr Very good. (0.4) very good=lemme see yer ankle. (2.2)

Dr Pt. hhh VERY GOOD

Pt I wanna ask yih som'n

Dr What's that. (Frankel 1990: 241)

The sequence above demonstrates one of the devices patients employ, a sequentially modified question, designed to manoeuver the interactional restrictions of the medical encounter. This operates, Frankel argues (in contrast to ordinary interaction), to reduce the force of asking a direct question by delaying the placement of that question in its initial position by, in this case, using a prefatory utterance (I wanna ask yih som'n). Such utterances are utilized by patients to seek permission to initiate the act of asking a question. Their use also provides some evidence of the patient's uncertainty about asking a question, its placement in the ongoing sequence of activities as well as how the doctor will respond.

Of course, since the 27 years following Frankel's work, this restrictive interactive form has come under increasing scrutiny as the patient‐centred approach, which is designed to foreground the patient's knowledge and experience as part of the interaction to effect active patient involvement, has steadily gained more momentum. Despite this, however, evidence can still be provided of patients’ dispreferences to ask questions in the consultation and this is not only limited to primary care encounters. More recently, evidence of the constraining effect of the structure of the medical encounter has been found in oncology consultations. In these settings, patients’ information needs are both varied and substantial,^11^ yet patients rarely ask questions unless explicitly invited to do so.^11^ Ford et al^12^ have shown that oncologists’ lack of explicit invitations to explore the patient's psychosocial state resulted in minimal opportunity for patients to express information needs and initiate discussion. McJannett et al^13^ report on recently diagnosed cancer patients feeling uncomfortable asking the surgeon questions about their diagnosis and its implications, because they felt uncertainty as to when to ask questions during the consultation.

To help encourage patients to ask questions, question prompt lists (QPLs) have been used extensively in oncology. QPLs are a list of questions that the patient can ask the doctor during the consultation to target their information needs and encourage more dialogical exchange between doctor and patient. A number of research studies have been conducted on the use of QPLs in oncology consultations, which indicate some success regarding patient question asking.^14^, ^15^ In particular, QPLs have been shown to have a positive influence encouraging question asking about diagnosis and prognosis.^16^, ^17^

There are, however, two fundamental issues that require further examination. Firstly, the definitive impact of QPLs on improving patient question asking has yet to be established.^18^ Findings from studies of QPLs tell us whether the activity (patient question asking) itself occurs, thus providing a measure of whether patient question asking was helped or hindered by the QPL. However, they generally do not provide us with detailed insight into which specific communication practices actually generate or create more interactional space for patient‐initiated questions. Without insight into the situationally specific and sequentially distinctive elements, which may have influenced the occurrence of patient questions in the first place, we are not in a good position to capitalize on the efficacy of the QPL. Even in cases where QPLs have been shown to increase patient question asking in relation to diagnosis,^17^ we have very little information on the precise nature of the doctor's communication behaviour (or any other communication factors), which may also have encouraged patient questions.

The second point is related to the first. Taking diagnosis as a case in point, evidence indicates that patients rarely provide any substantive response following a diagnostic announcement from the doctor.^10^, ^19^ This may in part be due to the fact that patients typically orient to diagnosis as the domain of clinical expertise.^10^ In relation to this, there is substantial evidence to suggest that news announcement and patient response are related and that the design of diagnostic news announcement can effect and shape patient response.^19^, ^20^, ^21^, ^22^

The findings reported in this paper examine the relation between the announcement of diagnostic news and patient question asking in oncology consultations. Conversation analysis (CA) is used to characterize the different ways in which diagnostic news was announced to highlight the sequential implications of different types of announcement on producing (or not producing) a patient question in the next turn of talk.

## 2 METHODS

### 2.1 Participants, setting and procedure

The findings are taken from the communication component of a larger study designed to investigate patient involvement in oncology consultations. Ethical approval for that larger study was obtained from Nottingham Research Ethics Committee 2 (reference number 09/H0408/34). Within that study 16 doctors were recruited (7 of 9 potential consultants and 9 out of 12 SPRs) of whom 6 were male and 10 were female. Patients who were new referrals to the department and those in follow‐up and undergoing active surveillance were recruited to explore their experiences of the consultation process. Patients had to know their cancer diagnosis, be over 18 years of age, and be willing to participate in the study. Our exclusion criteria included any patient unable to consent for themselves, patients with a cognitive impairment or patients who did not speak fluent English. Patients were sent a patient information sheet prior to their consultation, in the post inviting them to participate in the study, and those expressing an interest were seen by a member of the research team who provided further clarification if needed. Written informed consent was obtained from those doctors and patients willing to participate in the study.

182 patients were approached: 77 agreed to participate, 105 declined. 7 of the 77 withdrew consent, and 3 cases were incomplete. Of the remaining 67 participants, 47 had their consultations audio‐recorded. In each consultation, the audio recorder was placed on the desk and was switched on by either the researcher or the doctor conducting the consultation prior to commencing the consultation with the patient. From the primary sample of 47, we identified 30 consultations based on a basic count of the frequency of patient questions and their positioning in relation to diagnostic announcements of test results. This subcollection of 30 consultations were a mix of first and follow‐up consultations.

### 2.2 Analysis

All 30 consultations were analysed and transcribed (using transcription conventions from CA) by GM. As a sociological method, CA focuses on the sequential organization of talk enabling the identification of distinctive sequential structures. This method has been used widely in studies of doctor‐patient interaction in both primary and secondary care settings including oncology.^23^, ^24^, ^25^, ^26^ Each consultation was reviewed individually by GM, LF and AT, and notes were made. GM then compared observations to try to establish patterns of communication around patient question asking. This led to the identification of subcollections of patient question asking occurring more frequently in follow‐up consultations following the announcement of diagnostic news. However, in other follow‐up consultations patient questions did not arise following the diagnostic announcement. Closer inspection of these sequences was made to examine the potential relation between announcement and the occurrence of a patient‐initiated question in the next turn. Any disagreement regarding the interpretation of the data was resolved through continued discussion by all three authors. Extracts from eight of the follow‐up consultations (Table 1) are discussed below as they represent a further subcollection of the strongest examples of the relation between diagnostic announcement and patient question asking. All drafts and revision of the analysis were made by GM.

**Table 1:**
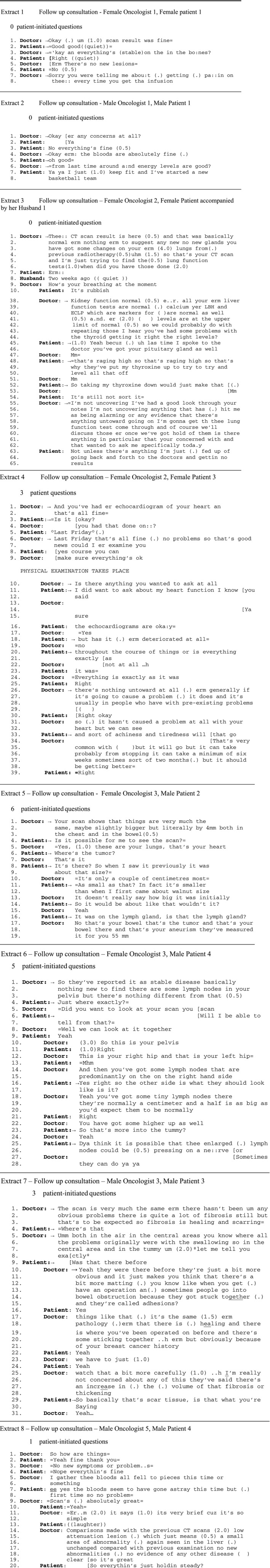
Patient question asking following the delivery of results

## 3 RESULTS

How diagnostic news was delivered is particularly important, as it seemed to create or close down opportunities for patient questions. We counted patient‐initiated questions immediately following the diagnostic announcement, as they could be directly attributable to the type of diagnostic announcement. We identified two ways in which diagnostic news was delivered, Q− and Q+.

### 3.1 Typical features of the Q− delivery

- A “no problem” general assessment/formulation (normal/fine).
- No problem formulation provides the upshot of findings potentially closing down further discussion of topic.
- Patient alignment (minimal response) with the “termination of topic” implicativeness of the formulation/general assessment.
- Minimal time allotted between patient response and doctor's next utterance.
- Next utterance (the doctor's) consisting of a) further general assessment or b) topic change.

We did not count patient‐initiated questions following the transition to another topic as these could not be directly connected to the type of diagnostic announcement. The Q‐ type of diagnostic announcement occurred in 18 consultations of the subcollection of 30. In each instance, no patient questions followed the diagnostic announcement.

### 3.2 Typical features of the Q+ delivery

- Announcement of the news almost immediately followed by a more detailed reporting or description of the news.
- A short pause following the delivery.
- Patient's response (typically a question).
- Further elaboration sometimes with visual representation of results (scan/x‐ray).
- Patients sometimes proposing their own interpretation of diagnostic news sometimes leading to subsequent patient questions.

As with Q−, we did not count patient‐initiated questions following the transition to another topic as these could not be directly connected to the type of diagnostic announcement. However, in some instances of the Q+ cases the invocation of the scan or X‐ray results provides space for further patient questions. This type of diagnostic announcement occurred in 12 consultations of the subcollection of 30.

### 3.3 The frequency and location of patient questions

The subcollection of 30 consultations in total came to 451.30 minutes, just over 7.5 hours of consultation time with the average length of the consultation at 15.04 minutes. In 7 of the 30 consultations (just under 60 minutes of consultation time), patients did not ask any questions. In the remaining 23 consultations, there were 76 instances of patients asking questions (average 2.5 direct patient questions per consultation). However, patients’ questions arose in different ways. For example, in 5 of those 23 consultations (22%) patients’ questions came at the end (within 3‐4 minutes of the end of the consultation) again following a prompt from the doctor. In 6 of the 23 consultations (26%), there is evidence of indirect or embedded questions arising at different junctures of the consultation following a prompt from the doctor. In 12 of the 23 consultations (follow‐up consultations) (52%), patient‐initiated direct questions occur following a diagnostic announcement. In 7 of these 12 consultations (58%), patient‐initiated assertive questions occur following a careful explanation of test results and diagnostic evidence. In only two consultations did the patient decline to ask a question following an invitation to do so from the doctor.

### 3.4 Extracts 1‐4

Extracts 1‐4 contain characteristics of the Q− format. In extract 1, there are no patient questions following the announcement of diagnostic results. At line 1, the doctor announces the scan result providing a general evaluation (“fine”), which is followed at line 2 by the patient's response “good.” The doctor provides another general assessment of the condition of the patient's bones at line 3. The patient aligns with this assessment at line 4. At line 5, the doctor changes topic to ask about the pain the patient has been experiencing.

At one level, extract 1 could be viewed as the patient deferring to clinical expertise with regard to the receipt of test results.^10^, ^19^ Within this sequence, there is minimal space for the patient to respond with no pause between the exchanges until line 6. There is some indication that the patient is orienting to upcoming news, as the utterances at lines 2, 4 and 6 are continuers giving the doctor the “go ahead signal.” Nevertheless, the form of news announcement and the minimal space between the patient's response and the next doctor's utterance appears to provide a more restricted sequential environment shaping minimal patient responses.

The news announcement in extract 1 is characteristic of a “no problem diagnosis”,^22^ in so far as the announcement projects the clinical cogency of the general assessment as to what the results mean. The absence of any specific detail in the doctor's delivery of results presupposes a level of information the patient requires. This projects a paternalistic mode where the patient is expected to rely on the authority of the medical interpretation.^21^

In addition, the form of the announcement emits a potentially limiting effect on the patient's response. It shares a characteristic of a formulation,^27^ a gloss for the practical management of the description of the findings. In this, and in other cases, the formulation “provides a constraint on the production of some next utterance”^27^ where in typically the patient's response is a confirmation of the general evaluation of findings. In effect, these general formulations of the findings perform “double duty”^27^ by providing the upshot of the news, whilst also acting to “close a topic down as a mentionable.”^27^ Consequently, there is a marked degree of separation between doctor and patient in relation to the entitlement to know, potentially resulting in a negative impact on patient question asking.

In extract 2, the doctor begins by asking whether the patient has any concerns. The patient's reply (“No everything's fine”—line 3) may be a consequence of two things. Firstly, the lexical choice of “any” when asking about concerns has been shown to minimize the expression of patient concerns.^28^ Secondly, the sequential position of this question, close to the start of the consultation, is quite telling for two reasons. At the start, the patient may be keen to progress the consultation in anticipation of diagnostic news foregoing any discussion of concerns at this point. Conversely, for the doctor, the placing of the question (“er any concerns at all”) at the start of the consultation, where there is no information at hand, has the potential to mitigate the risk of subsequent patient questions following the “no problem” diagnosis. That is to say, once the patient reports they have no concerns they may then find it difficult to raise a question later on when information (albeit in general form) is revealed.

The actual announcement of test results is given at line 4 and, in a similar way to extract 1, is accompanied by a general assessment/evaluation, (“absolutely fine”). Again the news is characteristic of a “no problem diagnosis”^22^ where the announcement projects the force of the general assessment concerning the meaning of the results. The patient accepts this (“oh good”—overlapping talk) as the doctor continues with the delivery of results. Following this, the doctor makes a transition to another topic asking about the patient's energy levels.

In both extracts, the patients can be seen as complicit in allowing the doctor to take the lead and shape the direction of the consultation. However, how the results are delivered is not incidental. The character of the news delivery in each case shapes the character of the consultation so that minimal interactional space is provided for patient question asking. This is even more apparent in extract 3.

In extract 3 at lines 1‐2, the CT scan result is announced with the evaluation “basically normal.” There is a suggestion of elaboration with the announcement of “no new glands” and “some changes on your lungs.” However, no further details are provided by the doctor, either on the status of the glands or the changes in the lungs, evidently a “no problem” diagnosis.^22^ The delivery of the results is terminated with “so that's your CT scan.” The doctor then makes a transition to another topic asking about the lung function tests.

Later in the consultation (at lines 38‐44), the patient is told that the kidney and liver function results are “normal” and calcium and other markers are “normal.” The doctor then (line 43) asks about the patient's thyroid. Following a 1‐second pause, the patient responds and in so doing demonstrates her understanding of the cause of the problem (pituitary gland is “raging high”) and the need to adjust the medication to return the pituitary gland to normal. In this response, the patient structures her involvement by attempting a move towards demonstrating independent expertise^21^ by showing the doctor her understanding of the causes and treatment of the thyroid problem. The patient's account demonstrates understanding by linking the symptoms to the reported facts (see the use of “because”), characteristic of an overt explanation.^24^

However, this demonstration of experiential knowledge is soon closed down by the doctor at lines 57‐58 with the clinical interpretation of the patient's condition, that there is nothing alarming going on. Indeed, throughout the patient's account (lines 45‐54), the doctor provides minimal responses, which could be read as “go ahead signals” allowing the patient to hold the floor. However, when the patient finishes (line 54), the doctor immediately comes in to gloss the account and close down any continued discussion of the patient's concerns regarding their current medication, (“I'm not uncovering I've had a good look through your notes I'm not uncovering anything that has (.) hit me as being alarming”). Notably, it was the doctor who opened up the topic (line 42), which led to the patient's account. At line 60, there is a prompt from the doctor for the patient to ask a question. This is declined as the patient expresses their discontent with (“going back and forth to the doctors and gettin no results”).

### 3.5 The “Deviant Case”

Extract 4 presents a possible deviant case. At lines 1‐2, the doctor announces the results of the echocardiogram. Unusually, following this general assessment (“fine”) the patient asks whether the results are okay (line 3). There is overlap here (lines 3‐4), and consequently, this question is overridden as the doctor and patient try to establish when the test was done. Following this, the doctor provides an evaluation of the results again “that's all fine, no problems.” Following the examination (line 10), the doctor provides a prompt creating interactional space by inviting the patient to ask a question. This is the only extract where the patient is invited to ask a question with the invitation occurring in relatively close proximity to the delivery of test results and following physical examination. However, even when given a clear opportunity to ask a question, the patient orients to the “deference structure” by producing a sequentially modified question.^9^ (“I did want to ask about my heart function”). The patient then targets back on the original evaluation of the results of the echocardiogram function, producing two further questions on possible deterioration and achiness and tiredness. In this case, although the “no problem” diagnosis is delivered at the start, the sequential positioning of the invitation for the patient to ask a question (in close proximity to the announcement and the physical examination) provides a possible different approach to encouraging patient questions.

#### 3.5.1 Extracts 5‐8

Extracts 5‐8 are more characteristic of the Q+ format described above. In extract 5, the sequence begins almost like the Q− delivery with the general evaluation, “things are very much the same.” However, the doctor follows this by describing the evidence “slightly bigger but literally by 4 mm both in the chest and bowel.” This reporting of the findings is immediately followed (at line 4) with the patient, unusually, requesting to see the scan. In so doing, the patient makes a very definite move towards gaining independent expertise by “actively pursuing information in accord with his own interests.”^21^ The doctor responds by showing the scan, which is then followed by a further six questions from the patient regarding the tumour, its size and the location of the lymph gland. Each question follows on from a piece of information delivered by the doctor regarding what the scan is showing. The critical factor in this extract appears to be the description or reporting of the evidence when the news is announced at the beginning. This contrasts with extracts 1‐4 where the doctor's own clinical assessment/evaluation (albeit general) of the findings is provided from the outset. The announcement alongside the reporting of particular information appears to encourage information‐seeking behaviour from the patient, which in this case is evident with the patient's request to see the scan.

A similar thing occurs in extract 6. Here, the delivery of diagnostic news opens with the reporting of the findings “so they've reported it as stable disease basically.” The utterance “stable” implies some assessment, but in fact, this is a feature of the report rather than the doctor independently providing an assessment of the findings to the patient. The doctor then goes on to report the fact that there “are some lymph nodes” in the patient's pelvis but “nothing different from that.” There is then a half second pause providing a space in the next turn for the patient's question “just where’ exactly” (line 4) as in extract 5 the patient now demonstrates an interest in gaining independent expertise.^21^ The doctor then asks the patient whether they would like to look at their scan. The patient's next question (“will I be able to tell from that”?) at line 6 orients to the asymmetrical nature of the encounter (discussed above) and is responded to by the doctor (“we can look at it together”) inviting the patient to examine the scan jointly. Again, the initial reporting of the findings, rather than the provision of a general assessment of the findings, appears to be a factor in shaping the patient's response in the next turn. Subsequently, a further three questions follow on from looking at the scan together culminating in the patient proffering their own assessment at line 25 based on the information they have received from the doctor.

Extract 7 shows that the doctor announces the results (“The scan is very much the same”) at line 1 but then follows this with an assessment, but with elaboration explaining the result and providing particular detail. The doctor continues by informing the patient that there is still fibrosis but that “is to be expected.” At line 4, the patient responds by asking where the fibrosis is. Because the audio data do not afford visual access, it is not clear from the recording whether the doctor shows the scan to the patient or not when explaining where the fibrosis is (lines 5‐8). The patient then asks whether the fibrosis was present before (line 9). The doctor confirms that it was there before and explains what fibrosis is (lines 12‐15, 17‐20) and places this in the context of the patient's breast cancer history (lines 20‐21). After giving their assessment of this (lines 25‐26), the doctor refers specifically to the report (lines 26‐27 “They've said there's an increase in the volume of that fibrosis.” Following this, the patient formulates^27^ the news (“that's scar tissue is that what you're saying.”) and by doing so makes a move towards gaining independent expertise,^21^ a move also evident in extracts 5 and 6.

Finally, extract 8 presents an interesting case as it consists of one patient question; however, it evidences features of both Q− and Q+ approaches, beginning first with Q− and then leading into Q+. Consequently, an evident contrast between the two is made immediately apparent. The Q− is delivery occurs at line 9 (Scan's (.) absolutely great), producing an immediate minimal response from the patient (line 10). At line 11, however, the doctor makes a transition to Q+ by reporting the actual scan results (“Er..m (2.0) it says”), which is completed at line 19 where the doctor repeats the initial general assessment having just reported and explained the actual findings. It is immediately followed at line 19 with the patient's question (So everything's just holdin steady?), which, as in extract 7, operates to formulate upshot of the news.

## 4 DISCUSSION

The findings from this study potentially provide some direction in addressing the uncertainty patients may experience in relation to when to ask a question in oncology consultations^13^ and potentially other consultation encounters. We have identified two ways in which diagnostic results are delivered, Q− and Q+. Each type of delivery appears to have different sequential implications for patient questing asking. The Q− delivery seems to produce a minimal response from the patient, whereas Q+ results in patient questions and generally more patient involvement subsequent to the announcement of the news. With the Q+ announcement, patients sometimes propose their own assessment of the diagnostic news in a move to gain independent expertise^21^ and fuller understanding.

The difference between the two types is quite subtle but significant. In Q−, the announcement is encapsulated with a clinical (albeit general) assessment (“your scan is fine”). Typically, this results in a minimal patient response, which may in part be due to the fact that implicit in Q− is the doctor's judgement about what constitutes sufficient information for the patient. Moreover, formulating the upshot of the news in this way can potentially “close a topic down as a mentionable.”^27^ In contrast, in Q+ the announcement consists of a description or reporting of the results sometimes without an explicit assessment. (“So they've reported it as stable”). This description or reading of the results without an evaluation “cast [s] patients as likely or possibly or capable of interpreting the reading.”^21^ This appears to be responded to by patients as a relevant position in the consultation for information seeking which in the cases discussed results in patient question asking.

These differences in announcing the news of results hold implications for the use of question prompt lists (QPL), which have been widely used and researched within oncology. The issue concerns the fact that the prompt itself may not be capitalized if patients do not feel an appropriate interactional space has been created to ask a question. The QPL may provide the patient with an agreed platform for question asking, but this does not always guarantee that the patient will ask questions. These findings indicate that with some basic training doctors may be able to secure patient involvement by providing an interactional space for patients to act on by asking questions in a way that is naturally occurring.

### 4.1 Study limitations

The study is limited by its sole reliance on audio recordings of consultations. Consequently, other aspects of social interaction, for example, eye contact, bodily comportment, which can also have a significant influence on the things like the provision of interactional space, have not been included. Moreover, there may be various reasons why patients were disinclined to ask questions following the Q− delivery. The character of patients’ responses may not actually be conditioned solely by the type of announcement of test results but may have more to do with patient preferences or information needs at these particular moments. Their responses could also be shaped by differences in the social or cultural background of the patients, their experience of the kind of cancer they have, their symptoms as well as any knowledge of prognostic outcome they may have acquired. Finally, the sample size is a relatively small one and a larger sample would perhaps give more in the way of generalizability of results.

## 5 CONCLUSION

How, when and under what circumstances patients are inclined/disinclined to ask questions is a complex issue. In our data, even in consultations where patients were asking questions, they tended to allow the doctor to take the lead with only one example of a patient asking a direct question without any pause or prefatory utterance following the doctor's utterance (extract 7 line 4). Q− approaches minimized the opportunities for patients to ask questions. Q+ approaches provided more scope for patient engagement, which on several occasions resulted in patients formulating the upshot of diagnostic news. Nevertheless, how patients respond to the delivery of news, may be shaped by other “presuppositional grounds.”^9^ Therefore, it may be premature at this stage to suggest a direct relation between delivery and response. It would, therefore, seem sensible to test the claims made in this paper to see whether they stand up to further investigation.

## INFORMED CONSENT AND PATIENT DETAILS

I confirm all patient/personal identifiers have been removed or disguised, so the patient/person(s) described are not identifiable and cannot be identified through the details of the story.

## CONFLICT OF INTEREST

None.

## APPENDIX 1

### Transcription symbols

**Table:**

º º	Talk marked by the degree sound indicates words that are softly spoken
(.)	A full stop in brackets indicates a micro pause
(1.0), (0.5)	Indicates silence in seconds and tenths of seconds
[Okay	
[Yes Talk which is preceded by a square bracket indicates overlap in speech between two different speakers	
=	Talk marked with the equals sign at the end of one line and the beginning of another indicates no pause between the end of one utterance and the start of another
::	Indicates prolonged sound
→	Indicates notable utterance
.	Indicates a falling, or final intonation contour, not necessarily the end of a sentence.
?	Indicates rising intonation not a question although in some instances the two occur together
,	Indicates continuing intonation.
